# Profile of Data Sharing in the Clinical Neurosciences

**DOI:** 10.7759/cureus.9927

**Published:** 2020-08-21

**Authors:** Keith A Hanson, Nyle Almeida, Jeffrey I Traylor, Dayal Rajagopalan, Jeremiah Johnson

**Affiliations:** 1 Department of Surgery, Texas Tech University Health Sciences Center, Lubbock, USA; 2 Department of Neurosurgery, University of Oklahoma School of Medicine, Oklahoma City, USA; 3 Department of Neurosurgery, University of Texas at Austin Dell Medical School, Austin, USA; 4 Department of Neurosurgery, Baylor College of Medicine, Houston, USA

**Keywords:** data sharing, open access

## Abstract

Importance: In today’s climate of high healthcare costs and limited research resources, much attention has been given to inefficiency in research. Open access to research data has been proposed as a way to pool resources and make the most of research funding while also promoting transparency and scientific rigor.

Objective: The clinical neurosciences stand to benefit greatly from the potential opportunities afforded by open data, and we sought to evaluate the current state of publicly available research findings and data sharing policies within the clinical neurosciences.

Design: The Clarivate Analytics Web of Science journal citation reports for 2017 were used to sort journals in the category ‘Clinical Neurosciences’ by impact factor. The top 50 journals were selected and reviewed, but data was only collected from journals focused on original research (42/50). For each journal we reviewed the 10 most recent original research articles for 2016, 2017, and 2018 as designated by Scopus.

Results: A data sharing policy existed for 60% (25/42) of the journals reviewed. Of the articles studied 41% (517/1255) contained source data, and the amount of articles with available source data increased from 2016 to 2018. Of all the articles reviewed, 49.4% (620/1255) were open access. Overall, 6.9% (87/1255) of articles had their source data accessible outside of the manuscript (e.g. registries, databases, etc.) and 8.9% (112/1255) addressed the availability of their source data within the publication itself. The availability of source data outside the manuscript and in-article discussion of source data availability both increased from 2016 to 2018. Only 3.9% (49/1255) of articles reviewed reported negative results for their primary outcome, and 7.6% (95/1255) of the articles could not be defined as primarily reporting positive or negative findings (characterization studies, census reporting, etc.). The distribution of negative versus positive results reported showed no significant trend over the years studied.

Conclusion and Relevance: Our results demonstrate an opportunity for increased data sharing in neuroscience original research. These findings also suggest a trend towards increased adoption of open data sharing policies among journals and increased availability of unprocessed data in publications. This can increase the quality and speed at which new research is developed in the clinical neurosciences.

## Introduction

Neuroscience is a field currently experiencing tremendous growth [1]. Significant technological advances in “omics” platforms and the advent of large-scale neuroscience research programs have generated vast amounts of primary data [2]. Of these ambitious data-gathering endeavors in the non-clinical setting, the Brain Research through Advancing Innovative Neurotechnologies (BRAIN) Initiative and Human Connectome Project (HCP) are among the most notable examples [3,4]. The BRAIN Initiative and HCP, along with other ambitious large-scale neuroscience research programs, have generated unprecedented primary data repositories and complex digital datasets that require further scientific analysis and dissemination [5]. Biomedical imaging modalities and computational methodologies have expanded in lock-step with the nascence of large-scale neuroscience research programs, further complicating an increasingly diversified pool of digitized primary data [6,7]. As the bulk of these data-sharing advancements are rooted in the non-clinical neurosciences, the lack of systematic data sharing in the clinical neurosciences has become a serious concern [8]. 

It is well established that data sharing is beneficial for the clinical neurosciences and other scientific disciplines as a whole [9]. The integration of complex computational and behavioral datasets would ameliorate the many challenges clinicians face with siloed primary data [10]. Implementation of standardized data-sharing policies by journal publishers stands to play a critical role in mitigating the problems of limited sample sizes, lack of transparency in reported outcomes, and multiple modes of bias that invariably affect the clinical neurosciences and other clinical research disciplines [8,11]. These intrinsic problems are further exacerbated by the growing presence of “long-tail” data and “dark” data [12,13]. Long-tail data, which encompasses a large portion of clinical research data, is data produced by studies that are small in scope with limited reproducibility [12]. Dark data refers to the negative results and primary data that remain unpublicized or are omitted due to their failure to validate hypotheses [13]. Dark data directly contributes to the misappropriation of limited research funds and the unnecessary repetition of studies, and these data types constitute an alarmingly high proportion of the overall data in neuroscience research [12,13]. 

The challenge of data sharing in neuroscience has not gone unrecognized. Funding agencies now frequently require data sharing or management policies for research grants and proposals [11,14]. Journal publishers have also increasingly adopted data sharing policies in recent years [9,11]. Standardized neuroscience data sharing formats are becoming a reality. For example, the Neurodata Without Borders (NWB) initiative led to the production of an integrated and easy-access Hierarchical Data Format 5 (HDF5)-based pilot model for neurophysiological metadata within a year [7]. Despite these successes, significant problems remain. While initiated by certain journal publishers, the implementation of data sharing policies is primarily left to the author’s discretion [11]. This results in marked variation in data sharing policies between journals [15]. Furthermore, there is limited oversight on journal data sharing policy compliance [8]. Once generated, shared data repositories often lack standardized maintenance for ensuring longevity and accessibility [16,17,18]. 

The state of data sharing for primary data in clinical neuroscience journals is currently unknown. We sought to elucidate prevailing data sharing practices for primary data in the clinical neurosciences from 2016 to 2018. This investigation offers insight into the reality of data sharing policies and their efficacy in the clinical neurosciences. Most importantly, these findings identify areas for improvement in neuroscience data sharing practice that could further enhance reproducibility, reduce waste, and possibly lead to the improvement of patient-centered outcomes.

## Materials and methods

To determine the journals used for analysis, a Clarivate Analytics Web of Science journal citation report was generated for 2018 (accessed August 2018). Results were filtered to only include those journals in the category ‘Clinical Neurosciences,’ and journals were sorted by impact factor. The top 50 journals by impact factor were reviewed. Of those journals, data was collected for 42 of the original 50 as only journals focusing on original research were included in the study. The list of studied journals and their associated impact factors can be seen in Table 1. Journals were evaluated for open access status and publicly available data sharing policy. 

**Table 1:** Journals evaluated Evaluated journals listed by impact factor with associated citation count and Eigenfactor score based on the Clarivate Analytics Web of Science journal citation reports for 2017 (accessed August 2018).

	Total Cites	Journal Impact Factor	Eigenfactor Score
Lancet Neurology	28,671	27.138	0.06904
Acta Neuropathologica	18,783	15.872	0.04149
Alzheimers & Dementia	10,423	12.74	0.03004
Jama Neurology	6,885	11.46	0.03527
Brain	52,061	10.84	0.07517
Annals of Neurology	37,251	10.244	0.05339
Neuro-Oncology	10,930	9.384	0.03035
Movement Disorders	26,511	8.324	0.03798
Translational Stroke Research	2,202	8.266	0.00526
Neurology	88,493	7.609	0.11553
Journal of Neurology Neurosurgery And Psychiatry	29,695	7.144	0.03298
Stroke	65,854	6.239	0.08852
Brain Pathology	4,952	6.187	0.00775
Brain Stimulation	4,263	6.12	0.01451
Neuropathology and Applied Neurobiology	3,654	6.059	0.00635
Neurotherapeutics	3,973	5.719	0.00898
Pain	36,132	5.559	0.038
Multiple Sclerosis Journal	10,675	5.28	0.02189
Sleep	20,547	5.135	0.02587
Epilepsia	26,301	5.067	0.03249
Alzheimers Research & Therapy	2,192	5.015	0.00847
Journal of Neurotrauma	14,508	5.002	0.02113
Journal of Pain	9,264	4.859	0.01689
Journal of Stroke	694	4.75	0.00288
Therapeutic Advances in Neurological Disorders	1,004	4.75	0.0028
Journal of Psychopharmacology	5,808	4.738	0.0109
Parkinsonism & Related Disorders	8,967	4.721	0.01991
Neurorehabilitation and Neural Repair	5,032	4.711	0.00985
Annals of Clinical and Translational Neurology	1,377	4.649	0.00645
European Journal of Neurology	10,206	4.621	0.01935
Bipolar Disorders	5,070	4.49	0.00787
Neurosurgery	28,592	4.475	0.02593
Journal of Neurosurgery	34,561	4.318	0.03075
Progress in Neuro-Psychopharmacology & Biological Psychiatry	9,823	4.185	0.01317
European Neuropsychopharmacology	6,920	4.129	0.01511
International Journal Of Neuropsychopharmacology	6,259	3.981	0.01455
Cephalalgia	8,721	3.882	0.01394
International Journal of Stroke	3,825	3.859	0.01488
Journal of Affective Disorders	26,957	3.786	0.05338
Journal of Neurology	14,359	3.783	0.02516
Neuroepidemiology	3,261	3.697	0.00564
American Journal of Neuroradiology	22,667	3.653	0.02984

For each chosen journal, the first 10 consecutive original research articles published in the years 2016, 2017, and 2018 were reviewed. Article selection was accomplished using Scopus (most accessed October 2018; two journals for 2018, Ann of Clin Neuro and Parkin and Related Disord, were accessed from Scopus in March 2019). The list of available documents for each journal was generated and filtered by the criteria ‘Article’ and the relevant year. Available documents were then sorted by date of publication, and any remaining meta-analyses, case reports, educational articles, and review articles were excluded from the final list. Data collected included open access status, the availability of source data, funding source, journal policy, and whether the article reported positive or negative results for their primary outcome measures. In some cases funding data could not be acquired due to language barriers, funding source website errors, etc. Also, author compliance with journal/funding source policy was evaluated based on the available policy at the time of review, regardless of year. While we recognize that articles from 2016 cannot comply with a policy established in 2017 or 2018, the data still provides a picture of how authors in the neurosciences have used and shared their source data over time. 

It is critical that we be clear on the nature of what we sought to evaluate. When reviewing articles, the authors defined source data as any data used in the generation of the study outcomes that had not been processed, mathematically or otherwise. For example, individual survey responses in a psychiatric study on depression symptoms would qualify as source data. A table presenting averages for the same data does not. A microscopy image taken from a single animal subject is source data- a composite image for a particular subgroup in the same study is not. Also, data regarding funding and journal policy was meant to be evaluated from the perspective of a curious author or researcher. As such, no direct contact was made with journal or funding source representatives when conducting this study. We report what was publicly available at the time of review, which took place from October 2018 to March 2019. Any p-values stated in the results are the product of a chi-squared analysis with a significance cutoff of p = 0.05. 

## Results

**Figure 1:**
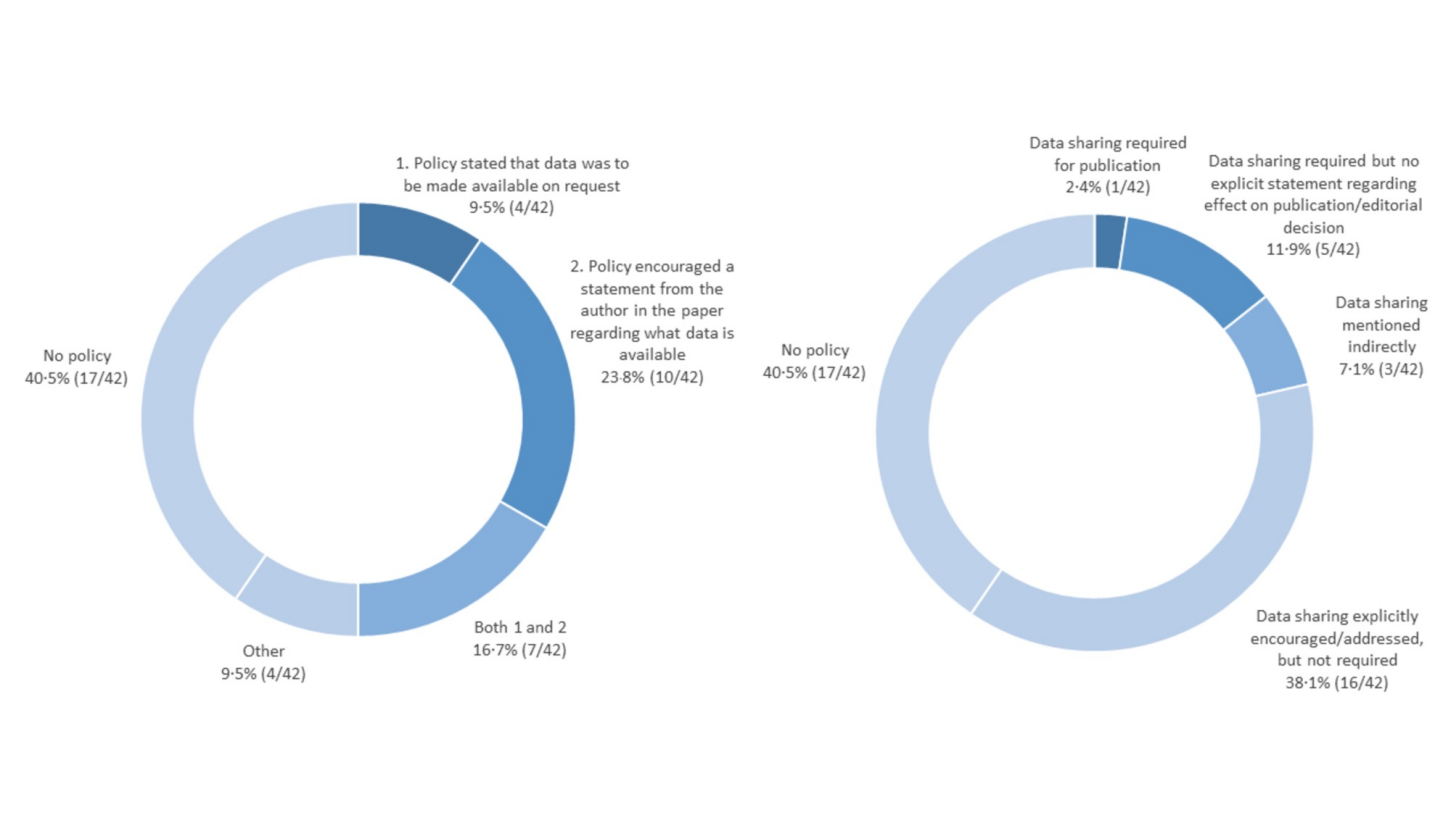
Journal policy distribution Distribution of journal policies based on the authors' original data sharing policy scale (Johnson et al.; left) and the policy scale based on the work of Vasilevskey et al., as cited by the European Commission’s facts and figures for open research data (right).

A total of 42 journals and 1255 articles were reviewed in this study. Of these journals, 60% (25/42) had an available stated data sharing policy at the time of review. The specific policy types and distributions are described in Figure 1. 

**Figure 2:**
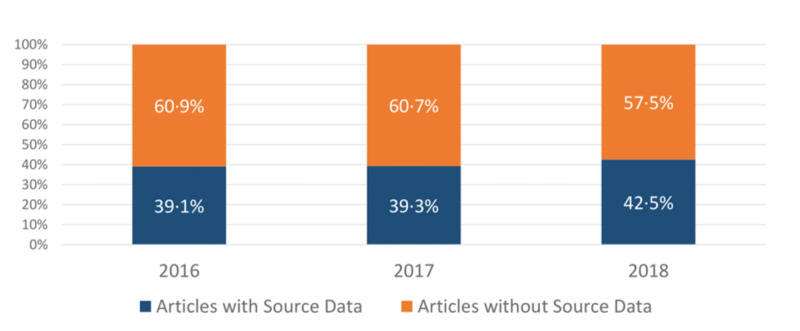
Article source availability Research article source data availability in 2016, 2017, and 2018. n = 417 articles for 2016 and 2017. n = 419 articles for 2018

Of the articles studied 41% (517/1255) contained source data, and the results (78%, 403/517) and supplementary (33%, 169/517) sections were the most common sites for such data. Please note that source data was often available in more than one section of the article. In addition, the availability of source data showed a positive trend from 2016 to 2018 as demonstrated in Figure 2. 

Regarding open access, 9.5% (4/42) of the reviewed journals were listed as open access. Of all the articles reviewed, 49.4% (620/1255) were open access, and the percentage of articles that were open access showed a negative trend over time. Overall, 6.9% (87/1255) had their source data accessible outside of the manuscript (e.g. registries, databases, etc.), 8.9% (112/1255) addressed the availability of their source data within the publication itself (e.g. data sharing statement), and 20.7% (151/727) of relevant manuscripts were compliant with their associated journal data policy. 

**Figure 3:**
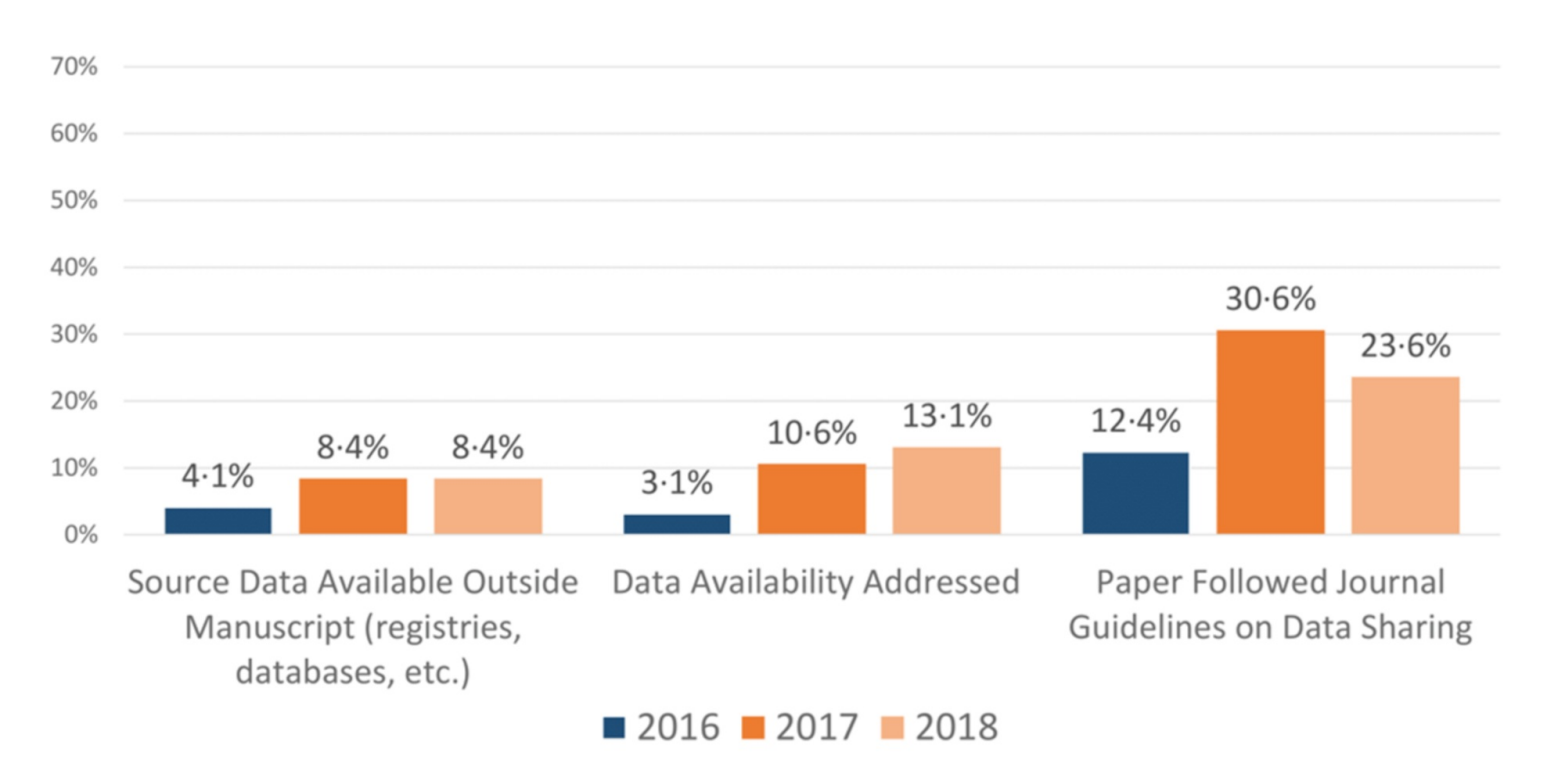
Data sharing trends Data sharing trends in 2016, 2017, and 2018. Captured trends include (1) the availability of source data outside the manuscript, (2) if data availability was addressed in the article, and (3) if the paper followed journal guidelines on data sharing. n = 417 articles for 2016, 2017 and n = 419 articles for 2018.

**Figure 4:**
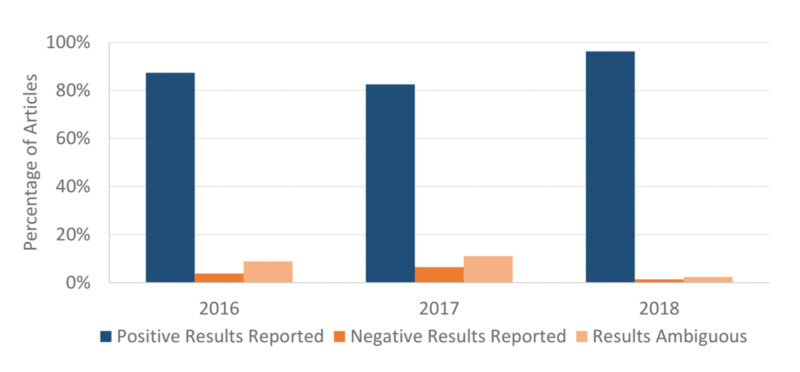
Article results Type of primary outcome results reported by manuscripts in 2016, 2017, and 2018. Positive, negative, and ambiguous results reported by percentage of total articles

As mentioned previously, there is a tendency for publishers and authors to only report positive results, and this contributes to bias, waste, and poor reproducibility. Part of open data best practice is the consistent sharing of negative data, so we also evaluated the conclusions (positive or negative) of the articles studied. Of the articles reviewed, 3.9% (49/1255) reported negative results for their primary outcome. 7.6% (95/1255) of the articles could not be defined as primarily reporting positive or negative findings (characterization studies, census reporting, etc.). The change in these variables over the years studied can be seen in Figures 3 and 4.

 

In addition, only 20.8% (121/757) of the reviewed articles followed the data sharing suggestions or requirements of their associated journal policy. However, articles funded by an organization with a data sharing policy were still more likely to share their data outside the manuscript (10.1% vs 6%; p < 0.05), discuss data availability within the manuscript itself (12.1% vs 8%; p < 0.05), and provide their article via open access (61.3% vs 45.7%; p < 0.05) when compared to unfunded research and research funded by an organization without a current data policy. Articles with an associated journal data policy were more likely to discuss their data availability within the manuscript (13.1% vs 2.8%; p < 0.05) and less likely to publish open access when compared to articles published in a journal without an associated data policy (44.2% vs 57.2%; p < 0.05). Journal data policy had no significant correlation to the availability of data outside of the manuscript. Of all articles reviewed, 64% (804/1255) listed a funding source specific to the project discussed in the article (author-specific funding was excluded), and 35.6% (286/804) of those articles were funded by an agency with an explicit data sharing policy.

## Discussion

The goal of this review was to evaluate the current state of data sharing in the clinical neurosciences. Our results demonstrate that data sharing in clinical neuroscience journals and manuscripts is increasing, but the overall rates of sharing source data remain low. Authors have become more willing (or are increasingly required) to publish or discuss the availability of their data when publishing manuscripts. Based on annualized rates, primary data, which includes source data present in supplementary repositories, is being shared more frequently. Despite the increase in data sharing over time, authors who actively share and discuss their source data are still a small minority. The majority of journals surveyed had an explicit data sharing policy, although the exact nature of policies varied greatly and compliance with the policies was limited. Compliance rates with journal policy trended upward over the years studied, but this change may be partly the result of our application of policies accessed in 2018 to articles published in 2016 and 2017. While funding sources with data sharing policies were in the minority, the funding agencies with such policies were often larger and more prevalent (e.g. National Institutes of Health, UK Medical Research Council, European Commission, etc.). Our findings also showed that articles backed by funding sources with data sharing policies were more likely to share data and make their articles freely available to the scientific community at large. Journal data sharing policies also correlated with increased discussion of data availability by authors. However, publications in journals with associated data sharing policies were less likely to be open access, and the presence of a journal data sharing policy was not associated with the availability of source data outside the manuscript. 

Varying rates of scientific reproducibility has led many to question whether scientists are in the midst of a reproducibility crisis. In a recent e-mail survey from Nature to approximately 1,500 scientists, it was revealed that 52% of respondents agree there is a “significant” crisis, with 38% agreeing there is a “slight” crisis [19]. The reasons for upwards of 90% of survey respondents believing that a reproducibility crisis exists is complex. However, most agree that the reproduction and validation of findings is made more difficult by variability in experiment design, absence of available raw data, pressure to publish, lack of information required to repeat a study, poor statistical power, and most importantly - selective reporting [19]. 

Our findings demonstrate a lack of negative results reporting in the clinical neurosciences that is consistent with the results of this survey. Our study also showed no significant improvement in negative results reporting over the years reviewed. In the current “publish or perish” culture, which seems to be especially prevalent in clinical fields, one cause of this discrepancy is clear. Researchers, especially those at the start of their career, have little incentive to share data or report on failed experiments and negative findings irrespective of how they may feel about data sharing in general. Reproducing studies often entails high material costs and further manual labor that contributes to the already glacial pace of most scientific research. Additional intangible costs are accrued in the form of a scientist’s time and career prospects, as the perception remains that the PI is not engaging in research that is their own. This research environment is not conducive to reproducibility, and it is reasonable to assume that scientists might forego reproducing experiments for the reasons described above. Increasing data sharing policies may result in the required paradigm shift, described by Koslow et al., necessary to fix the reproducibility crisis [20]. Increased availability of primary data, as well as recognition for the generation of such data, would invite greater sharing, discussion and clarification of both positive and negative results, thus ameliorating some of the obstacles that have led to the current reproducibility crisis. 

Limited funds exist in the clinical neurosciences, and lack of reproducibility and underreported negative findings result in the inadvertent, wasteful redundancy of research efforts [21,22]. There is also evidence to suggest that the availability of source data declines over time when confined to the investigator, further compounding challenges of waste and poor reproducibility [23]. From this perspective, increasing data sharing may offer funding agencies and national regulatory bodies greater transparency in highlighting research areas with superfluous or wasteful funding. Conversely, increasing data sharing would also provide an indirect avenue to identify funding “gaps” in previously unknown critical research areas, i.e. pinpointing overlooked topics in the clinical neurosciences. Tenopir et al. expound upon this duality of benefits of increasing data sharing from the perspective of funding agencies. Therefore it is no surprise that many large, public funding agencies in the United States, Europe, and Australia now require data sharing considerations, often in the form of a data management plan. International organizations, such as the International Committee of Medical Journal Editors, have also encouraged the sharing of clinical data in recent years [24]. Our findings support the effectiveness of such top-down policies in improving data sharing. However, further steps will have to be taken to support authors and ensure author participation as less than a third of all manuscripts reviewed followed their associated journal data policy in any of the years studied with an average compliance rate of approximately 20%. 

Our findings clearly demonstrate that authors in the clinical neurosciences are unable or unwilling to share and discuss their data, and it is important to recognize the numerous obstacles to sharing faced by the people who generate primary data. Apart from the aforementioned lack of incentives in publishing negative findings and cultural barriers in academics, significant costs exist. Generating, maintaining, and storing data is expensive and time-consuming, and proper data stewardship is not commonly taught to students at any level. Where is the best place to store data? How should it be formatted? Is the data safe from corruption? Will one be credited for the time and effort they have invested in procuring such data? In clinical fields, one may also ask whether it is even legal to share the data at all. These are all questions a researcher may pose when considering sharing their data, and unless extensive personal effort has been made by the individual researcher to educate themselves on the topic, it is unlikely that he or she will be able to answer these questions. However, there are tangible benefits associated with sharing data, ranging from increased citation metrics to publication in higher-profile journals [9]. For this reason, authors should consider the development of strong data sharing practices as a career asset. 

Fortunately, many organizations are taking steps to alleviate these concerns and knowledge gaps in an effort to promote data management best practices. The US National Academy of Sciences, Engineering, and Medicine (USNASEM) published a report, “Open Science by Design,” in 2018 which discusses ways universities, publishers, and other institutions can establish systems of reward for open data practices [25]. The report also highlights the importance of considering open data and science at every step of the research process. In conjunction with the European Commission, the USNASEM has endorsed a set of universal data management criteria called FAIR (findable, accessible, interoperable, and reusable), which can help guide researchers on how to best disseminate data [25]. In addition, organizations like OpenAIRE and FOSTER Plus have been created with the goal of educating researchers on open data practices [26,27]. Regarding costs and reward, some funding organizations, including the National Science Foundation, now allow research data and database costs to be included as allowable grant expenses [28]. The actual storage of research data is being made cheaper and simpler by non-profit organizations like Dryad, a public repository for research data run by publishers and scientific institutions, and re3data, an open registry of research data repositories across the sciences [29]. Other more unique methods to encourage data sharing have been attempted, including ‘web badges,’ created by the Center for Open Science, that researchers can use to label their articles and data as available and open. The introduction of such badge use by Psychological Science resulted in a 10-fold increase in the sharing of data from articles published within the journal following introduction of the badges in 2014 [30]. The use of such badges has been adopted by over 50 journals since their inception, implying that such approaches have the potential to be well-accepted in the scientific community [28]. 

Our review was subject to certain limitations that warrant discussion. While we attempted to be as rigorous as possible in our definition of source/primary data by establishing strict definitions prior to data collection, ultimately the wide variety of available published data types meant that the label of source vs. processed data was not entirely objective. Some data (e.g. pathology, radiology images) is more easily shared and classified as source data, while others (e.g. complex genomic data) proved more difficult to classify. As mentioned previously, in evaluating journal and funding source policy no contact was made with institutional representatives. It is therefore possible that a journal or funder did have a data sharing policy that the authors could not find. However, it is unlikely that such agencies contained a policy that could not be found in the associated website, author guidelines, and available publications. To add, we posit that since our goal was to evaluate open data practice and availability as it relates to the average researcher, anything that could not be found by our methods is likely ineffective and will be ignored by participating researchers. It should also be noted that the authors often struggled with the evaluation of some international funding sources. Language barriers and limited online resources often precluded rigorous review of such agencies, and these funding agencies were excluded from analysis. It is possible that the exclusion of such institutions, most often from China, South Korea, and Japan, could have skewed our results. To overcome this challenge, scientific journals and funding sources should make clear their policies on data sharing and access in the clinical neurosciences. 

It is important to reiterate that author compliance with journal and funding source policy was based on policies accessed in 2018-2019 even though the reviewed articles were published from 2016-2018. Especially when considering the delay from submission to publication, it is likely that some articles were submitted prior to implementation of their associated journal or funding source policies. While this makes any specific conclusions regarding author compliance less conclusive (as addressed previously), our data still allows for a picture of data sharing practice and the efficacy of data sharing policy and awareness over time. We know from our previous review on data sharing that at least seven of the 25 journals with a data policy in 2018 had a data policy in place in January 2016, and given that the highest yearly rate of compliance (30.6%, 2017) was well below a majority, it is safe to assume that our concerns regarding poor author compliance are warranted and our related findings are worth examining [17]. 

## Conclusions

Our findings suggest a positive trend towards increased adoption of open data policies among journals as well as a growing tendency among authors to share data. However, public availability of source data is still very limited, and in-article discussions of data access or availability are relatively rare in the literature. Reporting of negative results also remains rare in the clinical neurosciences, and this continues to complicate the current reproducibility crisis within medical science as a whole. No one party or institution is to blame for these concerns. In today’s research environment, there are many barriers, both cultural and concrete, to efficient data sharing and rigorous reproducibility. Authors are most likely to shoulder the cost via lost time, money, and productivity, often resulting in a lack of compliance with funding source or journal data policy. Despite the apparent growth of open data practices over time, the many fields that make up the clinical neurosciences will likely require more than simple policy requirements in order to overcome the many challenges associated with the availability and maintenance of open data.
